# Clock-linked genes underlie seasonal migratory timing in a diurnal raptor

**DOI:** 10.1098/rspb.2021.2507

**Published:** 2022-05-11

**Authors:** Christen M. Bossu, Julie A. Heath, Gregory S. Kaltenecker, Barbara Helm, Kristen C. Ruegg

**Affiliations:** ^1^ Biology Department, Colorado State University, Fort Collins, CO 80521, USA; ^2^ Center for Tropical Research, Institute of the Environment and Sustainability, University of California, Los Angeles, CA 90095, USA; ^3^ Raptor Research Center and Department of Biological Sciences, Boise State University, Boise, ID 83725, USA; ^4^ Intermountain Bird Observatory, Department of Biological Sciences, Boise State University, Boise, ID 83725, USA; ^5^ Department of Bird Migration, Swiss Ornithological Institute, 6204 Sempach, Switzerland

**Keywords:** genomics, seasonal migration, circannual rhythms, biological clock

## Abstract

Seasonal migration is a dynamic natural phenomenon that allows organisms to exploit favourable habitats across the annual cycle. While the morphological, physiological and behavioural changes associated with migratory behaviour are well characterized, the genetic basis of migration and its link to endogenous biological time-keeping pathways are poorly understood. Historically, genome-wide research has focused on genes of large effect, whereas many genes of small effect may work together to regulate complex traits like migratory behaviour. Here, we explicitly relax stringent outlier detection thresholds and, as a result, discover how multiple biological time-keeping genes are important to migratory timing in an iconic raptor species, the American kestrel (*Falco sparverius*). To validate the role of candidate loci in migratory timing, we genotyped kestrels captured across autumn migration and found significant associations between migratory timing and genetic variation in metabolic and light-input pathway genes that modulate biological clocks (*top1, phlpp1, cpne4* and *peak1)*. Further, we demonstrate that migrating individuals originated from a single panmictic source population, suggesting the existence of distinct early and late migratory genotypes (i.e. chronotypes). Overall, our results provide empirical support for the existence of a within-population-level polymorphism in genes underlying migratory timing in a diurnally migrating raptor.

## 1. Introduction

The annual migration of billions of birds is inherently linked to seasonal transitions across the globe. While it is generally accepted that birds migrate to reach favourable habitat for breeding or to avoid unfavourable conditions during the nonbreeding period [1–3], we are just now beginning to understand the underlying mechanisms that allow these model migratory vertebrates to detect seasonal cues that regulate migratory behaviour. Many species undergo major morphological, physiological and behavioural changes in preparation for migration [2,3], and these changes are often strikingly consistent in timing each year. Precise timing is achieved via a combination of environmental cue detection and endogenous biological clocks [4–6], with the exact interaction between the two biological clocks, circannual and circadian, remaining largely unresolved. While a core set of genes is known to regulate circadian clocks across vertebrate taxa [7–10], we are just starting to investigate the genetic basis of intraspecific variation in migratory behaviour. Given the importance of seasonal migration to adaptation across environmental gradients, a better understanding of the genetic factors underlying intraspecific variation in migratory timing will be important for predicting how animals will respond to future environmental change.

**Figure 1.**
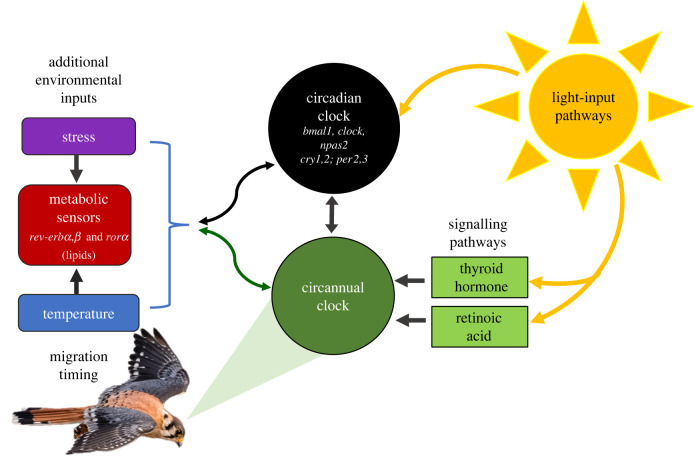
Avian migration timing is a complex interaction between the endogenous circadian and circannual clocks, synchronized primarily by light-input pathways, but also environmental inputs like stress, nutrients and temperature. Shown are the circannual clock (dark green circle) and its main signalling pathways (light green), along with the main physiological systems that act on it. These physiological systems link to sensors for important environmental inputs (yellow: light-input pathway; red: metabolic sensors; purple: stress; and blue: temperature). The circadian clock (black circle) has a regulatory role in these pathways and conversely also responds to them (two-ended arrows). Core clock genes known to be associated based on studies on model vertebrate systems are noted in each box. This study will investigate the potential for genetic associations within these core clock genes and migratory timing in American kestrels, as well as associations within less-well-described genes within environmental and metabolic input pathways that regulate the circadian and circannual clocks.

Past research supports the idea that the timing of morphological, behavioural and physiological changes associated with migration is partly genetically hardwired via circannual clocks with multiple environmental input pathways [4,6,11] (figure 1). For example, captive birds exposed to constant light : dark cycles show migratory activity (i.e. ‘Zugunruhe’ or migratory restlessness) that coincides with known migration of their free-living conspecifics, indicating that migratory timing is innate [6,12,13]. However, the timing of ‘Zugunruhe’ in captive birds starts to drift without adjustments to photoperiod, highlighting the importance of light-input pathways for correcting such drift (reviewed in [14,15]). In addition, metabolic sensors influenced by nutrients, temperature and stress are known to regulate the circannual timing of a suite of additional migratory traits, including fat deposition and orientation preference in songbirds [6,16–18]. On a phenotypic level, the interaction of environmental cues, like photoperiod, nutrient availability and temperature, with biological clocks can result in consistently early or late migratory phenotypes within a population, often defined as ‘chronotypes' [4,19]. Thus, while it is well recognized that both endogenous and environmentally controlled aspects of migratory behaviour are important, the genetic basis of distinct migratory chronotypes is yet to be fully explored.

While research on model organisms has facilitated a general understanding of the circadian clock system [20], research on the clock pathways that regulate seasonal migration in birds remains in its infancy [11,17,21]. Despite this, several key connections between clock pathways have emerged, which can inform the identification of genes that may underlie biological time-keeping in birds (figure 1). First, birds have a circadian system with multiple pacemakers that receive and decode photoperiodic information [22,23]. Circadian rhythms are generated by interlocking transcription–translation feedback loops and post-translational modification, driven by canonical clock genes [20,22,24], hereafter called ‘core’ clock genes (figure 1). Second, although the circadian system is mainly entrained by light, there is increasing evidence from mammal research that suggests temperature and stress sensors may also play a pivotal modulating role [25,26] (figure 1). Thus, core clock genes are regulated by ‘clock-linked’ genes involved in receiving and decoding photoperiodic and metabolic environmental cues. As a result, there is a need to investigate both core clock genes and clock-linked genes when investigating the genetic basis of migratory timing.

While many genes within clock pathways have been described, only a few have been strongly linked to phenological variability in animals (e.g. *clock*, *npas2* and *creb1* [7,9,10,27], but see [21]). Recent studies comparing populations of birds with divergent migratory phenotypes have succeeded in identifying a variety of genes putatively important to migration [28–32], but in most cases have fallen short in demonstrating functional links with the clock pathway [28,31]. The absence of consistent linkages between genetic variation in clock genes and phenological variability in birds may be due in part to the focus on core clock genes rather than genes that entrain and modulate the clock pathway, and/or to limitations in detection methods that focus only on highly significant outlier loci, thus ignoring genes of small effect [28–32]. Further, studies to date have largely focused on identifying genetic variation between populations with distinct migratory behaviours, rather than investigating the genetic basis of early and late migratory chronotypes within populations. To help fill these critical gaps in our knowledge, here we investigate the genetic mechanisms underlying distinct migratory chronotypes in a free-living migratory raptor, the American kestrel (*Falco sparverius*) using a combination of genome scans for candidate loci with relaxed detection thresholds and targeted genotyping of loci within clock and clock-linked genes.

There are a number of reasons why the American kestrel makes an excellent model for exploring the genetic basis of intraspecific variation in migratory behaviour. First, the American kestrel exhibits a wide array of migratory phenotypes across its North American range [33] (electronic supplementary material, table S1), from long-distance migrants in the north to short-distance migrants and residents (i.e. non-migratory) in the south [34], providing the opportunity for comparisons between populations with distinct migratory strategies. Second, a recent population genetic analysis within this species identified five main genetically distinct groups associated with migratory strategy (electronic supplementary material, figure S1)—three migratory and partially migratory populations to the north (western, eastern and Alaska) and two resident populations (Texas and Florida) [33]. Lastly, while American kestrel migration differs from songbird migration in many ways, there is support for the idea that migratory behaviour is heritable in migratory raptors [35] and thus may also have genetic components in kestrels. Here we take advantage of the American kestrel migratory system and knowledge of population genetic structure to further advance our understanding of the genetic factors regulating migratory timing.

We take an integrative approach to identifying the genetic basis of intraspecific variation in migratory timing that both reflects the polygenic nature of migratory phenotypes and accounts for the influence of population structure. To this end, we re-analyse a high-density restriction site-associated DNA (RAD)-sequencing dataset using an *F*_ST_-based analysis with relaxed detection thresholds to identify highly polymorphic core clock and clock-linked genes. We hypothesize that if migratory timing is controlled by many loci of small effect, then relaxed detection thresholds will identify potential candidate loci that may otherwise be missed using stringent thresholds. Based on the results of this analysis, we develop a suite of genetic assays to test the role of candidate loci in American kestrel migratory timing. We hypothesize that if genetic variation within core clock or clock-linked genes is important to regulating migratory timing in American kestrels, then we will find significant correlations between allele frequencies at these loci and migratory passage date. To test this hypothesis, we genotype 165 migrating American kestrels collected in a time series from a migratory station in Boise, Idaho. Lastly, to investigate whether correlations between allele frequency within target genes and migration timing are due to the presence of early and late migratory chronotypes within populations or genetically distinct populations migrating through at different times, we use population-specific genetic markers to identify the breeding population of migrants and investigate the correlation between allele frequencies in candidate loci and latitude. We predict that if associations between allele frequency and timing are due to chronotypes within populations then all migrating birds will be genetically identified as originating from a single panmictic source population and there will be no correlation between allele frequency and latitude.

## 2. Results

### (a) Identification of candidate migration-linked loci

We reanalysed a high-density RAD-sequencing dataset using *F*_ST_ outlier detection analyses [36] with relaxed thresholds to identify loci that differed between migratory/partially migratory and resident groups. Our analysis revealed 7227 polymorphic loci in 1843 genes (electronic supplementary material, figure S2). A subsequent literature search identified 21 of these genes with links to different aspects of seasonal migration (electronic supplementary material, table S3). These 21 candidate genes were grouped into four major categories, including (i) migratory timing (circannual and circadian rhythm, including metabolic sensors and photoperiodic pathways); (ii) morphological differentiation (i.e. cytoskeleton organization, muscle development and contraction, and bone metabolism which can increase bone density and strength); (iii) migratory restlessness (i.e. regulation of sleep and locomotor activity); and (iv) migrant physiology (i.e. lipid metabolism and increased fat storage) (table 1; electronic supplementary material, table S3). In order to investigate the role of a subset of these genes in migratory timing in American kestrels, we then designed genetic assays that could be used to rapidly screen polymorphic loci within the genes of interest. Because assay design for specific loci of interest is not always possible (see Methods), we were only able to design targeted assays for loci in 9 of the 21 total genes of interest. These included two core clock genes (*cry1, npas2;* figure 1), three clock-linked genes (*top1*, *cpne4* and *phlpp1*)*,* two genes linked to morphological differentiation potentially important to avian migration [28] (*lmbr1* and *nacc2*) and two genes (*peak1* and *scn5a*) known to be differentially expressed in the hypothalamus in Swainson's thrushes (*Catharus ustulatus*) during non-migratory and migratory states [32].

**Table 1.** Genes selected for validation by single-nucleotide polymorphism (SNP) genotyping in breeding and migrating American kestrels. The table includes gene name, hypothetical function, the candidate gene detection method that identified the gene, and details of the literature review and references.

gene name	function	details of function	references
*category 1—circannual and circadian rhythms, including metabolic sensors and photoperiodic pathways* (figure 1)			
*phlpp1*	circadian rhythm and light-input pathway	targeted deletion of the *phlpp1* gene in mice, where null mice display normal circadian rhythms, but an impaired capacity to stabilize the circadian period after light-induced resetting; involved in response of the circadian clock to light	[37]
*top1*	circadian rhythm and metabolism sensor pathway	mediates the impact of antagonistic metabolic sensors (RORs and REV-ERBs) on core clock genes; binds rhythmically to *bmal1* intermediate to two ROREs, thereby enabling ROR and REV-ERB action on *bmal1*; knockdown of *top1* expression lengthened circadian period	[38]
*npas2*	circadian rhythm pathway	core clock gene forming heterodimer with *bmal1* and regulated by metabolic sensors *rorα* and *rev-erbα*; *clock*-deficient mice with no functional *npas2* exhibited arrhythmic locomotor behaviour in constant darkness; found in several avian studies comparing migratory phenotypes	[8,39,40]
*cry1*	circadian rhythm pathway	CRY proteins function to repress BMAL1/CLOCK transcriptional activity to ensure the continuous daily rhythmic expression of genes	[20,41]
*nacc2*	circadian rhythm pathway	transcriptional repressor, may affect circadian rhythm in interaction with *mta1*; transcriptome sequence divergence in different willow warbler migration strategies	[28]
*category 2—traits related to migratory morphological performance with links to the clock pathways*			
*peak1*	cytoskeleton, cardiac and muscle regulation	pseudokinase involved in cell signalling and cytoskeleton organization, under regulation of EGFR and ERK pathway, which also affects circadian rhythm and light response, can feed back to EGFR*;* differential expression between migrant and non-migratory birds	[32,42,43]
*scn5a*	circadian regulated cardiac function	cardiac Na^+^ channel protein*. in* *vivo* and *in vitro* assays suggest that *scn5a* expression is under the regulation of the cardiomyocyte molecular clock, and a slower heart rate phenotype was observed in mice engineered to overexpress a dominant negative *clock* mutation in cardiomyocytes; differential expression between migrant and resident birds	[32,44]
*lmbr1*	limb development	*lmbr1* expression altered the developing limbs of *hemimelic extra-toes* (Hx) mice; the Hx mutation causes the loss or shortening of the radius and tibia and preaxial polydactyly on both forelimbs and hindlimbs; transcriptome sequence divergence in different willow warbler migration strategies	[28,45]
*category 3—migratory restlessness (i.e. regulation of sleep and locomotor activity)*			
*cpne4*	migratory sleeplessness	regulation of sleep, potentially memory consolidation and with ROR marker for claustrum in reptiles; differentially expressed in migratory versus non-migratory white-crowned sparrow	[46,47]

### (b) Testing the role of candidate genes in migratory timing

**Figure 2.**
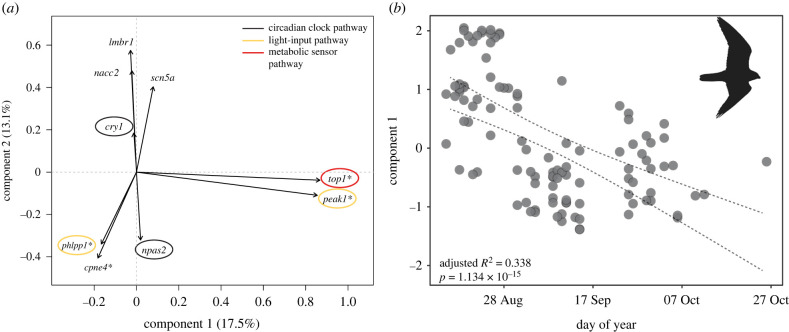
Validation of targeted candidate clock-, metabolic- and photic-linked genes with categorical PCA and linear regression of PC1 on passage date of migrant American kestrels. (*a*) Categorical PCA illustrates how potential clock-linked candidate genotypes covary in all genotyped birds. Colours represent the pathways the genes are associated with in figure 1 (circadian clock: black; light-input pathway: yellow; metabolic sensor pathway: red), and asterisks refer to the four genes that load substantially onto PC1. (*b*) The migratory candidate gene principal component 1 is significantly correlated to the day of year individual kestrels migrate through Idaho during autumn migration. Dotted lines indicate confidence interval.

A categorical principal component analysis (PCA) of genetic variation in the nine candidate migration-linked genes across all individuals (breeding and migratory) demonstrated high collinearity between certain genes (figure 2*a*). More specifically, PC1 explained 17.5% of the genetic variation and was weighted by variation in three clock-linked genes, *top1*, *cpne4* and *phlpp1*, and two genes associated with migration, *peak1* and *scn5a*. Alternatively, PC2 explained 13.1% of total genetic variation and was driven by variation in eight of the nine migration-associated genes, including the two core clock genes *cry1* and *npas2* (figure 2*a*; electronic supplementary material, table S2). Further regression analysis revealed a highly significant correlation between PC1 and autumn passage date in 165 individuals captured in the time series from an Idaho migration station (*p* = 1.134 × 10^−15^; figure 2*b*), but no correlation between PC2 and migratory passage date (*p* = 0.254, electronic supplementary material, figure S3). Overall these results support the idea that clock-linked genes involved in metabolic and light-input pathways are more important to regulating migratory timing than core clock genes.

Investigations into whether the association between PC1 and migratory timing was the result of migratory chronotypes within populations or genetically distinct populations migrating through at different times support the existence of distinct migratory chronotypes. More specifically, there was no association between PC1 and breeding latitude in western North America (*p* = 0.166; electronic supplementary material, figures S4B and S5A,C) and the majority of birds from the migration station in Idaho that could be assigned with certainty were assigned to a single panmictic population, the western genetic cluster (144 of 151 individuals). Further, the six individuals that were weakly assigned to the eastern cluster spanned early and late migratory periods, ruling out the idea that breeding origin may be confounding the relationship between migration timing and PC1. In addition, the effect of PC1 on autumn migration timing did not differ between females and males (*p* = 0.102; electronic supplementary material, figure S4A), and there was no significant relationship between sex and migration timing (*p* = 0.091), contrary to findings of sex-specific migration timing in other avian species [48,49].

**Figure 3.**
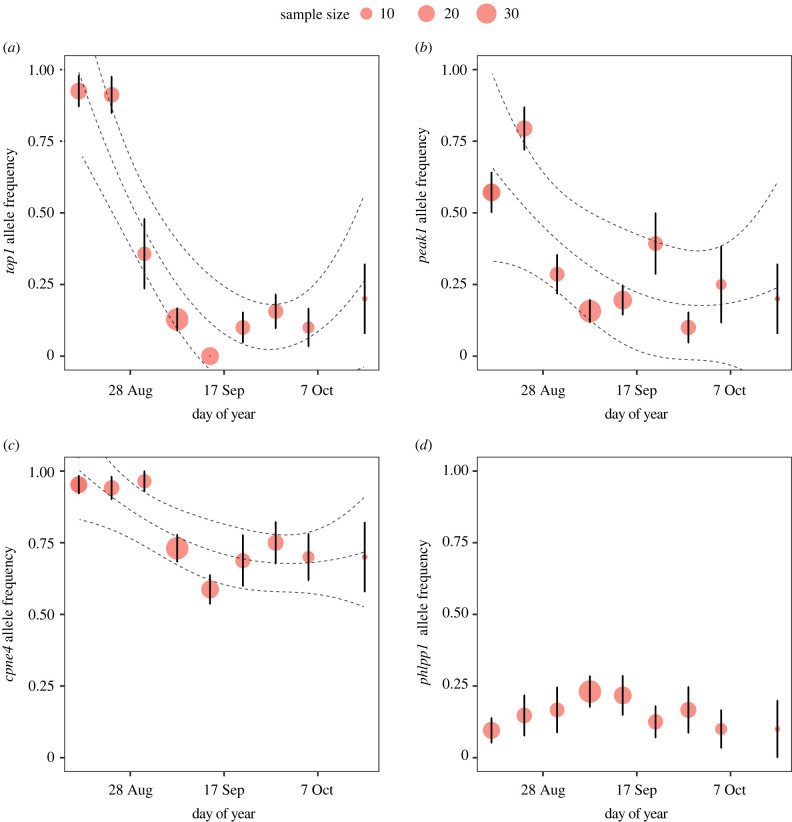
Allele frequency (i.e. proportion of major allele) of *top1, peak1, cpne4* and *phlpp1* (*a*, *b*, *c* and *d*, respectively) as a function of week during autumn migration (day of year) at an Idaho migration station. Point sizes are proportional to sample size (*n*) with the bars showing ±standard error of the mean (s.e.m.). (Online version in colour.)

Additional single-gene analyses further support the results from the multi-locus analyses and help elucidate which genes in particular are driving the observed patterns. The single-gene analyses demonstrated that allele frequencies in three of the top four genes that loaded highest on PC1, *top1*, *peak1* and *cpne4*, were strongly associated with migratory timing in a distinctly nonlinear fashion (*top1 p* < 2.2 × 10^−16^, *peak1 p* = 4.89 × 10^−09^ and *cpne4 p* = 2.98 × 10^−6^; figure 3). In particular, the observed correlations appear to be driven by a shift in allele frequency early in migration, as would be expected if early and late migratory chronotypes were passing through at different times. Further investigations into the potential influence of population-level effects on the observed patterns revealed that allele frequencies within each of these genes were not correlated to the breeding latitude of birds from across North America (see electronic supplementary material, figures S5B,D and S6). Overall, both the multi-locus and single-locus results support the hypothesis that genetic variation within clock-linked genes *top1*, *peak1*, *cpne4* and, to a lesser extent, *phlpp1* help regulate early and late migratory chronotypes in American kestrels.

## 3. Discussion

To investigate the genetic basis underlying migration, we combined a genome scan with targeted candidate gene analysis to document a significant correlation between genetic variation within metabolic and light-input pathway genes known to entrain circannual clocks and migratory timing in a free-living iconic raptor species, the American kestrel. Further, targeted genotyping revealed that genetic variation in three clock-linked genes was strongly associated with migrant passage date, but not breeding origin, supporting the idea that early and late migratory chronotypes exist within populations (figure 3; electronic supplementary material, figures S5B,D and S6). Overall, this work advances our understanding of the genetic complexities underlying migratory timing in a diurnal migratory raptor and provides, to the best of our knowledge, the first documentation of a genetic basis for early and late migratory chronotypes within populations of a migratory bird.

### (a) Clock-linked genes and migratory timing

While studies on a diversity of organisms have found significant associations between migratory timing and genetic variation in core clock-linked genes (*clock* and *npas2* [7,9,10,27]), here, we found no association between variation in two core clock genes, *npas2* and *cry1*, and migratory timing (electronic supplementary material, figure S7). While our result may seem counterintuitive, further information on the circadian clock feedback loops and the metabolic sensors and light pathways they interact with can help put our results into context [20,22,50]. For example, circadian rhythms underlying vertebrate locomotion, physiology, behaviour and gene expression are known to be controlled by a core set of genes that make up positive and negative feedback loops. In the positive limb of the feedback loop, *clock*, *npas2* and *bmal1* form a complex that activates the transcription of CRY and PER [24]. In the negative limb of the feedback loop, CRY and PER repress the transcriptional activity of the CLOCK/NPAS2–BMAL1 complex and facilitate daily rhythms in the expression of countless clock-controlled genes [20]. Thus, one explanation for the lack of an association between migratory timing genetic variation in core clock genes (*npas2* and *cry1*) found herein is that core genes remain highly conserved in order to preserve their central role in biological time-keeping and associated physiological processes [51–53].

By contrast, our results highlight a strong association between migratory timing and genetic variation in clock-linked genes (*top1*, and to a lesser extent, *phlpp1*) known to entrain the core clock pathway. More specifically, the CLOCK/NPAS2–BMAL1 complex promotes the transcription of metabolic sensors, RORs and REV-ERBs, known to fine-tune the circadian clock [54,55]. The gene with the strongest association with migratory timing in our study, *top1* (electronic supplementary material, table S2), is a key regulator of these metabolic sensors, such that knockout of *top1* results in lengthening of the circadian period in mice [38]. In turn, *phlpp1* plays a role in light-input pathways [37] such that its deletion in mice results in the inability to properly calibrate the circadian clock to light. Thus, while neither of these genes has previously been linked to migratory timing in birds, it makes sense that genetic variation within these genes could result in differences in period length, photic entrainment and subsequent seasonal timing. While more research is needed, our results highlight the potentially important role of genetic variation in metabolic and the light-input pathway genes (*top1* and *phlpp1)* to regulating seasonal timing in birds.

The links between the two other genes that were associated with migratory timing, *peak1* and *cpne4*, and biological clocks remain more tenuous. The gene *peak1* encodes a pseudokinase, and was previously found to be differentially expressed in thrushes in relation to migratory state [32], but no associations between *peak1* and biological clocks have been identified. One potential avenue for future work is to investigate the known associations between this gene and key signalling pathways that play a role in circadian and photic regulation [56–58]. For example, *peak1* is known to interact with ERK, a kinase that is involved in photic resetting of the clock in rodents [42,59], and is modulated by the retinoic acid signalling pathway (figure 1). Genetic changes in another kinase gene, *rock1*, were also recently found to be associated in Chinook salmon (*Oncorhynchus tshawytscha*) with run timing (e.g. spring versus autumn migration timing), as well as the timing of final ascent to spawning grounds in the Snake River [60,61]. In turn, *cpne4* has been associated with migratory restlessness in birds [46], and with sleep–wake cycle regulation, a core circadian process, in reptiles [47]. Its further association with memory function in mammals [62] might fit with recent reports that memory-linked genes are associated with long-distance migration in the peregrine falcon, *Falco peregrinus* [63].

Overall, our results support the idea that genes involved in metabolic modulation and entrainment of biological clocks, rather than core clock genes, play an important role in regulating autumn migratory timing in the American kestrel. These results are particularly important in light of the absence of information on factors controlling autumn migration in diurnal raptors relative to other more well-studied systems such as spring migration timing in nocturnally migrating birds or run timing in fish (e.g. [64]). Moreover, the high variation surrounding the association of PC1 and migration timing (figure 2*b*) suggests we have identified some genes of small effect, but are missing others and may not be accounting for potential epigenetic effects [65]. Future work will focus on investigating the mechanistic basis of the four genes discussed herein, as well as several additional genes with links to biological clocks identified by our broad-scale genomic analyses, but not specifically followed-up in migrating American kestrels (electronic supplementary material, table S3). In addition, repeating this analysis with whole-genome sequencing may reveal additional genes that contribute to the observed patterns.

### (b) Migratory chronotypes

While chronotypes often refer to variation in the timing of daily events between individuals, recent behavioural studies on birds have elucidated the link between the timing of daily events and the timing of seasonal migration at the phenotypic level [66]. Further research on birds and fish has demonstrated a link between circadian and circannual rhythms, where hormonal pathways triggered by a photoperiod signal, for example via melatonin and thyroid hormones, result in a seasonal phenotype [67,68] (figure 1). At the genotypic level, previous work on *clock* provided a tenuous link between genetic variation in genes central to daily rhythms and the timing of seasonal events, but this work was performed across rather than within populations [7]. Here, we document what is to the best of our knowledge the first example of an association between within-population-level genetic variation in genes that entrain biological clocks and the existence of early and late migratory chronotypes within populations (figure 3). Our conclusions are supported by allele frequency shifts (i.e. average population allele frequency over time) that were distinctly nonlinear in three of our top four migration-linked genes (*cpne4, peak1* and *top1*; figure 3). Further, most migrating birds in our study were genetically identified as originating from a single panmictic source population and no correlation was found between allele frequency and latitude, ruling out the possibility that early and late migrants represent birds from distinct geographic regions. Genetic evidence of seasonal migratory chronotypes in American kestrels provides some of the first support for the presence of an inherited migration programme in raptors.

In addition to helping unravel the genetic basis of migratory timing, the identification of a genetic polymorphism underlying early and late migratory chronotypes in American kestrels from western North America has importance for our understanding of how this population may or may not be able to shift migratory timing in the face of climate change [11,69]. Previous work has shown that the degree of standing genetic variation in migration-linked genes can have significant fitness consequences in rapidly changing environments. For example, work in European pied flycatchers (*Ficedula hypoleuca*) in The Netherlands suggested that the lack of genetic variation underlying the timing of spring migration constrained the advancement of breeding dates, despite the earlier onset of spring [70,71]. By contrast, a recent study of German hand-raised pied flycatchers suggested that the advancement of lay dates in wild populations over a 20-year period was almost completely explained by selection on the underlying circannual clock itself [72]. The existence of a polymorphism in migratory chronotypes within western populations of American kestrels suggests that this population has the raw material upon which natural selection can act to facilitate phenological shifts in the face of climate change. Future work on allelic diversity at these loci may help explain why populations in the west have advanced the timing of breeding in the past decade, whereas parallel reproductive advancements have not been documented in eastern North America [73].

## 4. Conclusion

Here, we used genome-wide reduced representation sequencing to identify significant associations between migratory timing in a diurnally migrating raptor and genetic variation in a suite of genes known to help regulate biological clocks across vertebrates. Our results support the idea that genetic variation in clock-linked, rather than core clock genes result in the existence of early and late migratory chronotypes within American kestrels from the western United States. Overall our results provide important insights into the factors controlling migratory timing in birds. Future work will focus on developing a better understanding of the linkages between climate-induced selection for phenological shifts, genetic variation in clock-linked genes and population declines in the iconic American kestrel.

## 5. Material and methods

### (a) Sample collection, DNA extraction and variant discovery

The genome-wide dataset used herein was previously used by Ruegg *et al.* [33] to assess patterns of population structure across the American kestrel breeding range. Because here we focus on questions related to the genetics of migratory timing, we only briefly describe the methods for sample collection and sequencing of the genome-wide data and refer readers to Ruegg *et al.* [33] for additional information. In short, in 2015 and 2016, we collaborated with several non-profit organizations, state agencies, university researchers and citizen scientists to sample 197 unrelated breeding adults or nestling American kestrels from 12 sites throughout the United States and Canadian breeding range. Of those populations with greater than four individuals, the subsequent genomic analyses focused on eight sites within the range of fully migratory and partially migratory American kestrels and two sites (Texas and Florida) that fell within the range of resident (i.e. non-migratory) American kestrels (electronic supplementary material, table S1 and figure S1). We extracted the DNA from the resulting samples using Qiagen DNeasy Blood and Tissue Kits and then used restriction site-associated DNA sequencing (RAD-Seq; [74]) to scan the genome for signals of selection across the breeding range. RAD sequences were aligned to an assembly of the American kestrel genome [33] and a total of 72 263 single-nucleotide polymorphisms (SNPs) were identified after quality filtering.

### (b) Identification of candidate migration-linked loci

To identify candidate loci associated with migratory behaviour, we used an *F*_ST_-based analysis with low detection thresholds. We created custom R scripts to identify loci with *F*_ST_ estimates that fell within a relaxed 90th percentile *F*_ST_ outlier threshold between the resident and migratory/partially migratory populations (Florida and Texas versus all other populations; electronic supplementary material, figure S2). Our threshold was intentionally low because our goal was not to identify highly significant outliers, but to identify many polymorphic loci of potential small effect that were linked to migratory behaviour. For one of our sampling sites (CA1; electronic supplementary material, table S1), it was unclear whether individuals were residents, migrants or partial migrants, and to avoid potential confusion this population was excluded from the *F*_ST_ analysis.

### (c) Testing the role of candidate loci in migratory timing

While many genome-wide association study (GWAS) approaches identify loci that are associated with complex phenotypes, these can include false positives, which prove misleading without subsequent functional validation studies that include, but are not limited to, functional assays, gene expression analyses and knockdown studies in model organisms (reviewed in [75]). In lieu of these studies, we designed Fluidigm SNP-type assays and screened additional breeding and migrating American kestrels that were independent of the RAD-seq analyses above. Specifically, we used the R package *snps2assays* [76] to evaluate the efficacy of designing assays for candidate loci. We considered the assays designable if GC content was less than 0.65, there were no insertions or deletions (indels) within 30 bp of the target variant, and there were no additional variants within 20 bp of the targeted variable site. We filtered out assays with primers that mapped to multiple locations in the genome (*bwa mem* [77]), resulting in assays for nine loci in nine candidate genes. We used the resulting Fluidigm assays to genotype the nine candidate migration genes in 738 breeding American kestrels from 83 sites and 165 migrating American kestrels from a single-migration station in Boise, Idaho collected in a three-month time series spanning autumn migration over 2 years (figure 2). For the migrating American kestrels, day of capture, sex of bird and band (a.k.a. ring) number were recorded.

We then used a multi-gene and single-gene framework to determine whether migratory timing was significantly associated with allele frequency shifts in the nine candidate migration genes. To determine how the nine candidate genes covary with each other, we conducted an ordinal PCA using the R software package *Gifi* [78] and found that PC1 explained 17.5% variation in the data (figure 2*a*). We used linear regression to broadly evaluate whether migration timing (day of year when an autumn migrant was captured) was associated with genetic variation as measured by PC1 and PC2 (figure 2*b*; electronic supplementary material, figure S3), and included a covariate of sex to account for the potential influence of differential migration between sexes on migration timing (electronic supplementary material, figure S4A). To investigate single-gene effects, we fitted linear regression models of each allele frequency of the top four candidate genes, i.e. those that loaded strongly on PC1, *top1*, *peak1*, *phlpp1* and *cpne4* (electronic supplementary material, table S2), to migration timing as defined by the midpoint day of each week during the autumn migration period (figure 3) and using the *lm* model in the R software package *stats* v. 3.6.2 [79]. The nonlinear decline in allele frequency over autumn migration of three of the top candidate genes, *top1*, *peak1* and *cpne4*, prompted the fitting of a curved regression model, and we tested whether this linear regression polynomial model provided a better fit using a likelihood ratio test in the R package *lmtest* v. 0.9-37 [80].

To test whether seasonal allele frequency trends result from different populations migrating through the migration station at different times or distinct migratory chronotypes, we examined the association between PC1 and latitude as well as allele frequency in our four top-ranked loci and latitude of kestrels breeding across the west (electronic supplementary material, figures S4B, S5 and S6). Further, we genotyped 151 of the 165 migrating birds from Boise, Idaho (all samples for which we had high-quality DNA remaining) with population-specific SNP-type assays used in Ruegg *et al*. [33] and assigned these birds to the breeding population of origin using *rubias* [81].

## Data Availability

The raw RADseq genomic data reported in this paper can be found on Dryad [82]. The raw genotype data and custom scripts are available on GitHub [83] (https://github.com/cbossu/AMKE_MigrGenomics) and Dryad (doi:10.5068/D1B69N). The data are provided in the electronic supplementary material.
